# Effects of low-dose milk protein supplementation following low-to-moderate intensity exercise training on muscle mass in healthy older adults: a randomized placebo-controlled trial

**DOI:** 10.1007/s00394-020-02302-4

**Published:** 2020-06-10

**Authors:** Kyosuke Nakayama, Yuri Saito, Chiaki Sanbongi, Koichiro Murata, Tadasu Urashima

**Affiliations:** 1 R & D Division, Meiji Co., Ltd., 1-29-1 Nanakuni, Hachioji, Tokyo 192-0919 Japan; 2 Department of Human Sciences, Obihiro University of Agriculture and Veterinary Medicine, Inada-cho, Obihiro, Hokkaido 080-8555 Japan; 3 Department of Food Science, Obihiro University of Agriculture and Veterinary Medicine, Inada-cho, Obihiro, Hokkaido 080-8555 Japan

**Keywords:** Sarcopenia, Aging, Elderly, Hypertrophy, Lean body mass, Physical performance

## Abstract

**Purpose:**

The purpose of this study was to examine whether long-term ingestion of low-dose milk protein supplementation causes a greater increase in muscle mass and strength of older adults during low-to-moderate intensity exercise training intervention than isocaloric carbohydrate.

**Methods:**

In a randomized, double-blind, and placebo-controlled design, 122 healthy older adults (60–84 year) received either an acidified milk protein drink containing 10 g of milk protein (MILK; *n* = 61) or an isocaloric placebo drink (PLA; *n* = 61) daily throughout 6 months of body weight and medicine ball exercise training. Measurements before and after the intervention included body composition, physical performance and blood biochemistry.

**Results:**

Lean body mass significantly increased in the MILK group (+ 0.54 kg, *p* < 0.001), but did not change in the PLA group (− 0.10 kg, *p* = 0.534). The increases in the MILK group were significantly greater than in the PLA group (*p* = 0.004). Fat mass (− 0.77 kg) and plasma uric acid levels (− 0.3 mg/dL) significantly decreased only in the MILK group (*p* < 0.001), with a significant group difference (*p* = 0.002 and *p* < 0.001, respectively). Most of the physical performance tests significantly improved in both groups, but no group differences were found.

**Conclusion:**

We conclude that low-dose milk protein supplementation (10 g of protein/day) combined with low-to-moderate intensity exercise training is associated with increased muscle mass, but not improved physical performance compared to carbohydrate combined with exercise in healthy older adults. This study was registered in the UMIN Clinical Trials Registry (UMIN000032189).

**Electronic supplementary material:**

The online version of this article (10.1007/s00394-020-02302-4) contains supplementary material, which is available to authorized users.

## Introduction

Peak muscle mass occurs between the ages of 20 and 40 years, and then naturally declines as one ages, accelerating in older adults [1]. Sarcopenia, a marked decline in muscle mass in older adults, contributes to loss of independence because the loss of muscle mass reduces strength and functional capacity, both of which are needed to perform one’s activities of daily living. The loss of muscle mass is also associated with risk of diabetes [2] or heart disease [3]. Sarcopenia is a global problem for society, so it is important to develop effective strategies to maintain or increase muscle mass in older adults to combat sarcopenia.

Protein supplementation combined with resistance training may be an effective intervention to increase muscle mass regardless of age [4–7]. According to previous studies in older adults, a large dose of protein supplementation (> 20 g/day) increases muscle mass with [8–13] or without [14–16] resistance training. However, as the dose of protein increases, the size of the supplementation (foods or drinks) become bigger, and that may be a burden on older adults. Lower amounts of protein supplementation can ease the burden and can be incorporated easily into a daily routine, but the effects of low-dose protein intake (≤ 20 g/day) on muscle mass in older adults is unclear.

To detect the positive effects of low-dose protein supplementation on muscle mass in older adults, we designed the present study focusing on three points: high compliance, a longer intervention period, and high quality protein supplementation. First, we selected low-to-moderate intensity exercise training to maintain high compliance and minimize dropout. Second, we selected 6 months as the intervention period. Previous studies intervened for 10 weeks or more to detect the positive effects of a large dose of protein supplementation (> 20 g/day). In the present study, we set the protein dose to 10 g/day, less than half of the previous study design. We assumed that at least 20 weeks were required, so we set the intervention period to 6 months (26 weeks). Finally, we selected the acidified bovine milk we recently developed [17] as the protein supplementation. In a previous animal study, we showed that acidified milk had a greater effect on post-exercise muscle protein synthesis compared with skim milk, although both kinds of milk contained the same amount of bovine milk protein [17]. Bovine milk proteins are of the highest quality because they possess a complete profile of essential amino acids [18], have high amino acid absorptivity [19, 20], and stimulate muscle protein synthesis [21, 22]. The acidified milk might be more effective to increase muscle mass than normal bovine milk protein, such as skim milk, so we selected acidified milk as the intervention in the present study.

We examined whether 6 months of continuous ingestion of low-dose (10 g/day) milk protein supplementation caused a greater increase in muscle mass, strength and function in older adults compared with an isocaloric carbohydrate placebo drink during low-to-moderate intensity exercise training intervention.

## Methods

### Study design

The present study used a double-blind, placebo-controlled, parallel-group, randomized design. After acceptance into the study, participants were randomly allocated to either of two supplemented groups. All participants then started a 6-month exercise and supplement intervention. Before and after the intervention, body composition assessment, physical performance tests and blood sampling were performed. The trial was conducted at Obihiro University of Agriculture and Veterinary.

Medicine (Hokkaido, Japan) between February and November 2018. The data were analyzed between December 2018 and March 2019. The target number of participants was set at 128 (i.e., 64 participants per group) to detect a difference of 0.5 kg in the lean body mass change [6], assuming a standard deviation of 1.0 kg [11], to attain 80% statistical power using a two-sided *α* of 0.05.

### Participants

Participants were recruited from senior citizens’ clubs in Obihiro, Hokkaido, Japan. All participants completed health history and physical activity questionnaires and met the following inclusion criteria: 60 years or older and physically independent. Individuals were excluded if they (1) had current or previous histories of significant liver, cardiovascular, pulmonary, renal or digestive diseases; (2) had significant orthopedic injuries; (3) had food allergies; (4) restricted protein intake due to medical reasons; (5) were involved in regular resistance training (> 2 times/week); (6) were participating in other clinical studies; (7) or were judged as inappropriate for the study by the principal investigator due to abnormal blood pressure or parameters, or other reasons.

### Randomization and blinding

Stratification based on sex and age was performed after eligibility assessment for study participation. Participants were divided into four strata: (1) males less than 70 years old; (2) females less than 70 years old; (3) males 70 years or older; (4) females 70 years or older. In each strata, computer-generated random numbers were assigned to the participants who were then sorted and divided into two equal groups. The groups were randomly assigned to either the acidified milk (MILK) group or placebo (PLA) group, by an individual who was accountable for preparing the test drinks but was not involved in the plan, enrollment, evaluation, intervention, or analysis. The participants, investigators, and all staff members involved in the trial were blinded to group allocation. The randomization code was opened after the study data were checked, collated, and finalized.

### Interventions

#### Supplementation protocol

Participants ingested a 200 mL polyethylene terephthalate bottle drink daily of either an acidified milk protein drink [17] or a placebo drink for 6 months. The acidified milk protein drink was made from milk protein concentrate, trehalose, soybean polysaccharide, pectin, fermented cellulose, citric acid, malic acid and food flavors, and contained 7.0 g of carbohydrate, 10.1 g of protein and 0.2 g of fat, providing 68 kcal of energy per bottle. The amino acid profile of the acidified milk protein drink is shown in Table 1. The placebo drink was made from maltodextrin, trehalose, soybean polysaccharide, pectin, fermented cellulose, citric acid, malic acid and food flavors, and contained 16.0 g of carbohydrate, 0.1 g of protein and 0.5 g of fat, providing 68 kcal of energy per bottle. The test drinks had an identical pH (4.2) and appearance. The participants were instructed to keep the test drinks in their refrigerator and consume one bottle per day within an hour after each exercise training session. If the participants did not perform exercise training for some reason, they were instructed to consume a test drink anytime they wanted.

**Table 1:** Amino acid profile of the acidified milk protein drink

Amino acids	g/200 mL acidified milk
Alanine	0.37
Arginine	0.36
Asparagine + Aspartic acid	0.80
Cysteine	0.05
Glutamine + Glutamic acid	1.86
Glycine	0.20
Histidine	0.25
Isoleucine	0.50
Leucine	0.95
Lysine	0.69
Methionine	0.23
Phenylalanine	0.44
Proline	0.96
Serine	0.54
Threonine	0.44
Tryptophan	0.12
Tyrosine	0.52
Valine	0.73

#### Exercise training program

The exercise training program is shown in Table 2. All participants were instructed to perform an exercise training program daily for 6 months. The exercise training program was composed of 6 body weight exercises and 5 medicine ball exercises. The criteria for using low-to-moderate intensity exercise training [23] was that the participants were able to repeat the exercises at least 12 times (< 70% of 1-repetition maximum [24]). Part of the program was changed every 2 months to maintain participants’ motivation. Participants followed a monthly exercise training lesson throughout the 6-month intervention period (seven lessons in total).

**Table 2:** Exercise training program

0–2 months		2–4 months		4–6 months	
Bodyweight exercises	Reps	Bodyweight exercises	Reps	Bodyweight exercises	Reps
Standing calf raise	20	Single leg calf raise	10	Single leg calf raise	10
Squat	20	Side step squat	20	Squat	20
Abdominal crunch	10	Abdominal crunch	10	Abdominal crunch	10
Hip bridge	10	Single leg bridge	10	Standing leg curl	16
Side leg raise	10	Single leg raise	10	Single leg raise	10
Push up	10	Push up	10	Push up	10
Medicine ball exercises	Reps	Medicine ball exercises	Reps	Medicine ball exercises	Reps
Release and catch	30	Release and catch (half-rising)	20	Release and catch	30
Release and catch (with step)	20	Release and catch (with step)	20	Release and catch (with step)	20
Biceps curl	20	Biceps curl	30	Biceps curl	30
Upper body rotation	10	Upper body rotation	10	Upper body rotation	10
Leg flexion and extension	10	Leg flexion and extension	10	Leg flexion and extension	10

For the medicine ball exercises, participants used one or two 1 kg soft medicine balls.

*Release and catch*: Participants stood on the floor holding a medicine ball in front of their body in one hand. They released the ball and immediately caught the ball in their opposite hand with the palm facing down. They repeated the motion a given number of times (Table 2).

*Biceps curl*: Participants stood on the floor holding a medicine ball in each hand with their arms hanging by their sides. They performed a biceps curl a given number of times (Table 2).

*Upper body rotation*: Participants stood on the floor holding a medicine ball in front of their body in each hand. Then they rotated their upper body once with outstretched arms. They repeated the motion 10 times alternating from clockwise to counterclockwise.

*Leg flexion and extension*: Participants sat on the floor and put a medicine ball between their feet with their knees bent and their feet flat on the floor. They extended and bent their legs without dropping the ball. They repeated the motion 10 times.

#### Physical activity

Participants counted and recorded the number of steps daily using a triaxial accelerometer (FB-736, TANITA corporation, Tokyo, Japan) during the intervention period.

### Measurements

Body composition assessment, physical performance tests and blood sampling were performed at baseline and after 6 months of intervention by experienced staff members who were blinded to group allocation. On the day before the measurement, participants were barred from drinking alcohol and had dinner between 6 and 10 PM. After the dinner, participants were allowed to drink only water until the measurements started.

#### Body composition assessments

A direct segmental multifrequency (5 kHz, 50 kHz and 250 kHz) bioelectrical impedance analysis (DSM-BIA) device using an 8-point tactile electrode system (InBody 430, Biospace, Seoul, Korea) [25, 26] was used to measure body weight, lean body mass and fat mass. Participants were measured wearing the same shirt and pair of shorts, which weighed 0.6 kg, so the weight adjustment for clothing was set to 0.6 kg. Body composition assessments were performed between 8.30 and 11:30 AM in fasted states before physical performance tests.

#### Physical performance tests

On the measurement days, participants practiced each test twice with submaximal efforts to minimize the learning effect before making the actual test. They practiced the push-up motion twice.

*Grip strength*: A digital handgrip dynamometer (Grip-D, Takei Scientific Instruments Co. Ltd., Niigata, Japan) was used to measure grip strength. Subjects were allowed to adjust the grip on the apparatus, and then performed a maximum force grip with the right hand (isometric exercise) while the left arm was hanging free by the side. The test was carried out twice at an interval of 1 min and the best result was recorded as the grip strength.

*Maximal walking speed*: Participants walked on a flat, straight, 7-m-long walkway two times at their maximum speed. The time to walk 5-m was measured using diffuse-reflective photoelectric sensors (Yagami Inc., Aichi, Japan) which were placed at the 1-m and 6-m points. For maximum walking speed, the faster time recorded was used and converted into speed (m/s).

*Knee extension and flexion strength*: Knee extension and flexion strength were evaluated using an isokinetic dynamometer (Biodex System 4, Biodex Medical Systems, Inc. NY, USA) at an angular speed of 60°/s. Full knee flexion (start-position) was set to 100° and full knee extension was set to 30°. Following a familiarizing practice trial, participants extended and then flexed their right knees with full strength while crossing their arms in front of their chests. The test was repeated 3 times at an interval of 30 s and the best result was recorded as the knee extension and flexion strength.

*Timed Up and Go test*: The Timed Up and Go test measures speed during several functional maneuvers, which include standing up, walking, and turning and sitting down. The test is a reliable and valid test for quantifying functional mobility in older adults [27]. Participants were seated in a normal chair (41 cm high) with their back against the chair and their hands on their thighs. They were instructed to stand up, walk 3-m as quickly and safely as possible, past a cone on the floor, turn around, walk back to the chair, and sit down with back against the chair again [28]. A stop-watch was used to time one test. The test was carried out twice and the best time was recorded.

*Sit-to-stand test*: The sit-to-stand is often used as a measure of lower-limb strength in older adults [29]. The test measures the time taken to stand from a seated position either one, three, five or 10 times. In this study, participants were seated in a normal chair (41 cm high) and rose from the chair five times as fast as possible while crossing their arms in front of their chests. A stopwatch was used to time the test. The test was carried out twice and the best time was recorded.

*Push-up*: Participants fully extended their elbows and placed hands on the exercise mat directly under the shoulders, with knees together also touching the mat and trunk and thighs fully stretched. From this position, the elbows were flexed until just touching the mat with their chest and then immediately extended again. They repeated the push-up as many times as possible, and the number of repetitions was recorded.

#### Blood biochemistry

A blood sample was drawn from each participant in a fasted state at baseline. After 6 months of intervention, blood sampling was performed twice; the first sampling was performed in a fasted state, and the second sampling was performed 30 min after ingestion of 200 mL of the test drink (either an acidified milk or a carbohydrate drink). Immediately before ingestion of the test drink, participants performed the exercise training program (4–6 month version as described in Table 2). The second blood sampling was performed after body composition assessments and before physical performance tests. Plasma leucine was measured using liquid chromatography tandem-mass spectrometry (ACQUITY TQD, Waters Corporation, MA, USA). Other blood parameters were assayed by an independent laboratory (BML, Inc., Tokyo, Japan). Plasma albumin was measured using a bromocresol green method (Clinimate ALB, Sekisui Medical Co., Ltd., Tokyo, Japan). Plasma creatinine (Sikarikid-S CRE, Kanto Chemical Co., Inc., Tokyo Japan), uric acid (Pureauto S UA, Sekisui Medical Co., Ltd., Tokyo, Japan), low-density lipoprotein cholesterol (LDL-C) (Cholestest LDL, Sekisui Medical Co., Ltd., Tokyo, Japan), high-density lipoprotein cholesterol (HDL-C) (Cholestest N HDL, Sekisui Medical Co., Ltd., Tokyo, Japan) and triglycerides (Pureauto S TG-N, Sekisui Medical Co., Ltd., Tokyo, Japan) were measured using enzymatic methods. Plasma glucose was measured using a hexokinase assay (Pureauto S GLU, Sekisui Medical Co., Ltd., Tokyo, Japan). Plasma insulin was measured using a chemiluminescence immunoassay (Chemilumi Insulin, Siemens Healthcare Diagnostics, Inc., Tokyo, Japan). Plasma insulin-like growth factor-1 (IGF-1) was measured using an electro chemiluminescence immunoassay (Elecsys IGF-1, Roche Diagnostics K.K., Tokyo, Japan).

#### Dietary analysis

Before and during the 3rd and 6th month of the intervention period, participants recorded total food consumption for 3 consecutive days to determine their daily macronutrient intake exclusive of the test drinks. Food quantities were measured by using standard measuring glasses, spoons, and digital scales. All dietary data were analyzed by an independent laboratory (THF Co., Ltd., Ibaraki, Japan) using Excel Eiyo-kun, version 7.0 (Kenpakusha Co., Ltd., Tokyo, Japan).

### Statistical analysis

All statistical analyses were performed by an independent organization (edihas K.K., Hokkaido, Japan) using IBM SPSS Statistics 24 (IBM Japan, Ltd., Tokyo, Japan). Analyses were done on the full analysis set (FAS) with missing values imputed by the last observation carried forward. All values are expressed as mean ± standard error of the mean (SEM). The Student *t*-test for independent samples was used to compare differences between groups in participant characteristics at baseline, compliance rate for exercise training and consumption of the test drinks, daily average of the number of steps, and changes of values during the intervention period. A two-factor, repeated-measures analysis of variance (ANOVA) with time as the within-participants factor and group as the between-participants factor were carried out for dietary intake, body composition, physical performance, and blood parameters. When the F-ratio was significant, the Bonferroni post hoc test was employed to identify mean differences. For all statistical analyses, significance was set at *p* < 0.05.

## Result

### Participants

**Fig. 1:**
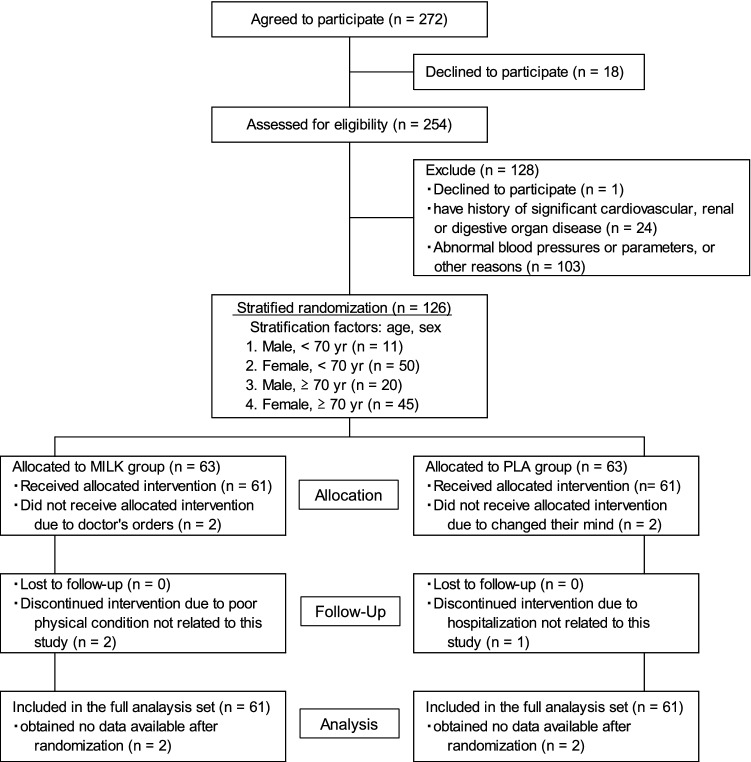
CONSORT flowchart for the human intervention study

The participant flow through the protocol is shown in Fig. 1. Written informed consent was obtained from 272 participants. Eighteen participants declined to participate in this study before the screening examination and 254 participants were screened. Finally, 126 participants were included in the study and randomly allocated to the MILK (*n* = 63) or PLA (*n* = 63) group. Four participants did not receive an allocated intervention due to a doctor’s orders (MILK = 2) or they changed their mind (PLA = 2); 122 participants (60–84 year, MILK = 61, PLA = 61) received the allocated intervention. Three participants discontinued the intervention due to poor physical condition (MILK = 2) or hospitalization (PLA = 1) not related to the study, so 119 participants completed the 6-month intervention. According to the FAS principle, the 122 participants (MILK = 61, PLA = 61) who began the allocated intervention were included in the analysis. The representative characteristics of the participants measured at baseline are presented in Table 3. There were no significant differences in any study outcomes between the two groups at baseline. No differences in the mean number of steps daily during the 6-month intervention period were observed (MILK: 6426 ± 299 steps/d, PLA: 6970 ± 326 steps/d, *P* = 0.220).

**Table 3:** Baseline participant characteristics

	MILK (*n* = 61)	PLA (*n* = 61)	*p*
Female, *n* (%)	45 (74)	47 (77)	–
Age, year	71.4 (0.8)	70.4 (0.7)	0.366
Height, cm	155.9 (1.1)	155.7 (1.0)	0.878
Body mass, kg	56.4 (1.3)	55.6 (1.3)	0.704
BMI, kg/m^2^	23.1 (0.4)	22.8 (0.4)	0.582
Body fat, %	29.0 (1.0)	27.7 (1.0)	0.363

Data are presented as mean (SEM)

*BMI* body mass index

### Dietary intake

Dietary intake (except for test drinks) before and during the 3rd and 6th month of the intervention period is presented in Table 4. During the intervention period, the total energy, protein and carbohydrate intake levels did not change but the fat intake significantly increased in both groups. There were no significant differences between groups in any dietary intake levels.

**Table 4:** Daily dietary intake (except for test drinks) before and during the intervention period

	MILK (*n* = 61)			PLA (*n* = 61)			*p* (ANOVA)		
	Before	3 months	6 months	Before	3 months	6 months	Time	Group	Interaction
Total energy, kcal/day	1864 (43)	1863 (48)	1906 (45)	1763 (39)	1803 (49)	1826 (50)	0.164	0.155	0.767
Protein, g/day	72.6 (1.9)	73.0 (2.0)	73.1 (2.1)	68.9 (1.9)	70.5 (1.9)	70.0 (2.1)	0.723	0.191	0.896
Fat, g/day	59.3 (2.0)	60.1 (2.3)	63.7 (2.5)*	54.7 (1.6)	57.7 (2.1)	60.8 (2.3)*	0.003	0.186	0.750
Carbohydrate, g/day	240.7 (5.9)	238.9 (5.8)	244.5 (5.8)	238.3 (6.0)	240.2 (6.8)	242.0 (6.9)	0.547	0.872	0.858

Data are presented as mean (SEM)

*Significantly different from before the intervention

### Protocol compliance

All participants followed the first exercise training lesson. There were no significant differences between groups in lesson compliance (MILK: 93.0%, PLA: 95.3%, *p* = 0.295), exercise training compliance (MILK: 92.6%, PLA: 92.0%, *p* = 0.790), or supplementation intake compliance (MILK: 96.4%, PLA: 97.3%, *p* = 0.577).

### Body composition

**Fig. 2:**
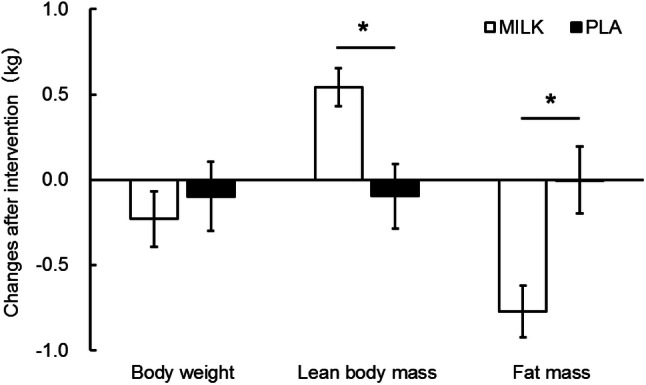
Changes in body weight, lean body mass, and fat mass after the intervention (6 months). Data are presented as mean ± SEM. *Significant differences between MILK and PLA

Body weight, lean body mass and fat mass before and after the intervention are presented in Table 5. Significant time × treatment interactions were found in the mean lean body mass and fat mass. Lean body mass significantly increased in the MILK group (+ 0.54 ± 0.11 kg, *p* < 0.001), but did not change in the PLA group (− 0.10 ± 0.19 kg, *p* = 0.534), following 6 months of the intervention (Fig. 2). The increases in lean body mass during the intervention period in the MILK group were significantly greater than those in the PLA group (*p* = 0.004). Fat mass significantly decreased in the MILK group (− 0.77 ± 0.15 kg, *p* < 0.001), but not in the PLA group (0.00 ± 0.20 kg, *p* = 0.990), following 6 months of the intervention (Fig. 2). The decreases in fat mass during the intervention period in the MILK group were significantly greater than those in the PLA group (*p* = 0.002). There were no significant changes or group differences in total body mass.

**Table 5:** Body composition before and after the intervention (6 months)

	MILK (*n* = 61)		PLA (*n* = 61)		*p* (ANOVA)		
	Before	After	Before	After	Time	Group	Interaction
Body weight, kg	56.4 (1.3)	56.1 (1.3)	55.6 (1.3)	55.5 (1.3)	0.206	0.693	0.609
Lean body mass, kg	39.7 (0.9)	40.3 (0.9)*	39.9 (1.0)	39.8 (0.9)	0.044	0.924	0.004
Fat mass, kg	16.6 (0.8)	15.8 (0.7)*	15.6 (0.8)	15.6 (0.8)	0.002	0.585	0.002

Data are presented as mean (SEM)

*Significantly different from before the intervention

### Physical performance tests

Physical performance test values before and after the intervention are presented in Table 6. Maximal walking speed, knee extension or flexion strength, Timed Up and Go test time, sit-to-stand test time, and push-up repetitions improved in both groups following 6 months of intervention (*p* < 0.001), but there were no significant differences between groups. Grip strength did not improve in either group following 6 months of the intervention.

**Table 6:** Physical performance test values before and after intervention (6 months)

	MILK (*n* = 61)			PLA (*n* = 61)			*p* (ANOVA)		
	Before	After	Change	Before	After	Change	Time	Group	Interaction
Grip strength, kg	27.3 (0.9)	27.4 (0.9)	0.0 (0.2)	28.1 (0.9)	27.5 (0.9)	− 0.6 (0.3)	0.176	0.721	0.132
Maximal walking speed, m/s	1.82 (0.04)	1.99 (0.04)*	0.17 (0.03)	1.90 (0.03)	2.03 (0.03)*	0.13 (0.02)	< 0.001	0.223	0.342
Knee extension strength, Nm	81.3 (3.0)	89.7 (3.2)*	8.4 (1.8)	80.9 (2.9)	91.1 (3.4)*	10.2 (1.4)	< 0.001	0.896	0.427
Knee flexion strength, Nm	44.1 (2.0)	49.0 (1.9)*	4.8 (0.8)	43.1 (1.8)	48.6 (1.9)*	5.4 (0.9)	< 0.001	0.790	0.613
Timed Up and Go test, s	5.83 (0.10)	5.55 (0.10)*	− 0.28 (0.07)	5.82 (0.10)	5.59 (0.09)*	− 0.24 (0.07)	< 0.001	0.894	0.631
Sit-to-stand test, s	7.86 (0.23)	6.93 (0.20)*	− 0.93 (0.15)	7.69 (0.19)	7.00 (0.18)*	− 0.69 (0.15)	< 0.001	0.844	0.262
Push-up, reps	14.7 (1.4)	22.5 (1.5)*	7.9 (1.4)	14.1 (1.6)	20.7 (1.8)*	6.6 (1.4)	< 0.001	0.555	0.535

Data are presented as mean (SEM)

*Significantly different from before the intervention

### Blood biochemistry

Fasting blood parameters before and after the intervention are presented in Table 7. There were significant time × treatment interactions in plasma creatinine and uric acid levels. Plasma creatinine significantly increased in the PLA group (*p* = 0.012) but did not change in the MILK group (*p* = 0.338) following 6 months of the intervention. There was a significant difference between groups in the changes in plasma creatinine levels (*p* = 0.014). Plasma uric acid significantly decreased in the MILK group (*p* < 0.001) but did not change in the PLA group (*p* = 0.528) following 6 months of the intervention. There was a significant difference between groups in the change in plasma uric acid levels (*p* < 0.001). Plasma IGF-1 in the MILK group (*p* = 0.043), HDL-C in the PLA group (*p* = 0.023), and triglyceride levels in both groups (MILK: *p* = 0.014, PLA: *p* = 0.045) significantly increased following 6 months of the intervention.

**Table 7:** Fasting blood parameters before and after intervention (6 months)

	MILK (*n* = 61)			PLA (*n* = 61)			*p* (ANOVA)		
	Before	After	Change	Before	After	Change	Time	Group	Interaction
Albumin, g/L	42.6 (0.3)	42.9 (0.3)	0.3 (0.2)	43.0 (0.3)	43.0 (0.3)	0.0 (0.2)	0.345	0.471	0.291
Creatinine, mg/dL	0.69 (0.02)	0.69 (0.02)	− 0.01 (0.01)†	0.70 (0.02)	0.71 (0.02)*	0.01 (0.01)	0.264	0.423	0.014
Uric acid, mg/dL	5.1 (0.1)	4.8 (0.2)*	− 0.3 (0.0)†	4.9 (0.1)	5.0 (0.1)	0.0 (0.1)	0.008	0.977	< 0.001
Glucose, mg/dL	95.3 (1.6)	94.2 (1.7)	− 1.1 (0.8)	94.2 (1.2)	93.5 (1.3)	− 0.7 (1.0)	0.165	0.669	0.780
Insulin, μU/mL	5.0 (0.6)	5.3 (0.5)	0.3 (0.4)	4.3 (0.5)	4.7 (0.3)	0.4 (0.5)	0.291	0.276	0.898
IGF-1, ng/mL	117.0 (3.5)	120.9 (3.7)*	3.9 (2.1)	109.2 (3.4)	111.5 (3.7)	2.3 (1.7)	0.023	0.083	0.561
LDL-C, mg/dL	128.1 (3.6)	124.9 (3.1)	− 3.2 (2.3)	125.2 (4.4)	127.1 (4.3)	1.9 (3.8)	0.774	0.945	0.251
HDL-C, mg/dL	70.5 (1.8)	71.4 (1.9)	0.9 (0.9)	70.0 (2.0)	72.1 (2.2)*	2.2 (1.0)	0.023	0.988	0.344
Triglyceride, mg/dL	83.8 (5.5)	93.6 (6.1)*	9.9 (3.0)	85.3 (4.8)	93.4 (5.9)*	8.0 (4.8)	0.002	0.932	0.746

Data are presented as mean (SEM)

*Significantly different from before the intervention

^†^Significantly different from PLA

**Fig. 3:**
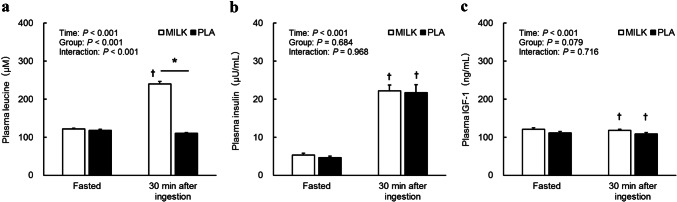
Plasma leucine (**a**), insulin (**b**), and IGF-1 (**c**) 30 min after ingestion of test drink following an exercise training session. *Significant differences between MILK and PLA. ^†^Significantly different from Fasted

Blood parameters 30 min after a post-exercise ingestion of 200 mL of the test drink are presented in Fig. 3a–c. Plasma leucine levels in the MILK group significantly increased 30 min after exercise and ingestion compared to a fasted state (Fig. 3a). The mean value of the plasma leucine increase was 119 ± 6 μM in the MILK group. In both groups, plasma insulin significantly increased (Fig. 3b) and plasma IGF-1 significantly decreased (Fig. 3c) 30 min after exercise and ingestion compared to a fasted state, with no difference between groups.

## Discussion

In this study, we found that post-exercise acidified milk protein supplementation throughout 6 months of low-to-moderate intensity exercise training (body weight exercises and medicine ball exercises) increased lean body mass and decreased fat mass in healthy older men and women compared with isocaloric carbohydrate supplementation. To our knowledge, this is the first study to demonstrate that the combination of low-dose milk protein supplementation (10 g of protein/day) and low-to-moderate intensity exercise training is beneficial to increase muscle mass in healthy older adults.

Many previous studies have investigated the effects of protein supplementation during exercise training on muscle mass in older adults. With adequate protein intake (> 20 g/day), the positive effects of protein supplementation on muscle mass have been reported frequently [8–13], but not always [30, 31]. At lower doses (≤ 20 g/day), significant effects of protein supplementation on muscle mass have not been found in any previous studies in exercised older adults [32–38]. In the present study, low-dose protein supplementation (10 g/day) was associated with increasing lean body mass in older adults, which is inconsistent with results in the previous studies. We propose three reasons for this. First, we speculate that exercise training intensity is associated with the effects of protein supplementation on muscle mass. It is well-known that high-intensity resistance training induces greater muscle protein synthesis [39] and muscle mass gain [40–42] compared with low-intensity resistance training. Most of the previous studies combined protein supplementation with high-intensity resistance training using weight machines or free weight training equipment (e.g., barbells) [8, 10, 12, 13, 30–32, 34–38], and those studies, except for one [13], reported a significant training effect on muscle mass without protein supplementation. In the present study, we selected low-to-moderate intensity exercise training to maintain high compliance and minimize dropout, and a significant training effect on muscle mass was not found. Two previous studies chose low (walking) [11] or moderate (body weight training and band training) [9] intensity exercise intervention, and significant protein supplementation effects on muscle mass were found although significant exercise effects were not. So it is possible that the large effects of high-intensity training obscured the effects of low-dose protein supplementation on muscle mass in the previous studies. Second, long-term, daily and high compliance protein supplementation increased total protein supplementation intake, and this may contribute to the gain of muscle mass. Although the daily supplementation dose in the present study was low, the total protein supplementation intake during 6 months was greater (approx. 1800 g) than in other low dose studies (< 1300 g) [32–35, 37, 38]. Krause [33] showed that a combination of low-dose protein intake (approx. 12 g/day) and low-to-moderate intensity exercise training, similar to conditions in our study, did not cause further muscle mass increase compared with the exercise-only group. That may be due to lower total protein intake (approx. 1000 g) during the intervention period or simply due to a shorter intervention period (12 weeks) than in the present study. Finally, the acidified milk protein drink, which was ingested in the present study, may have the potential to stimulate muscle protein synthesis more than other protein supplementations. We previously showed in an animal study that the acidified milk ingestion is associated with greater stimulation of post-exercise muscle protein synthesis compared with skim milk [17], although skim milk protein is also associated with marked changes in muscle protein synthesis [21] and muscle mass [43]. The acidified milk protein drink induced an acute increase in plasma essential amino acids, including leucine. Leucine is a potent activator of muscle protein synthesis [44, 45], and elevation of plasma leucine is associated with muscle protein synthesis stimulation: the ‘leucine trigger’ hypothesis states that there may be a threshold level of plasma leucine to trigger muscle protein synthesis [46]. Aging would increase the threshold [47, 48], so a large increase in plasma leucine levels is required to stimulate muscle protein synthesis in older adults. The acidified milk protein drink would meet the requirement in spite of the low protein dose in the present study. Actually, the mean value of plasma leucine increase (119 ± 6 μM) in the MILK group after ingestion of the acidified milk protein drink was comparable to previous studies [49, 50], which shows positive effects of protein source ingestion on muscle protein anabolic response in older adults. However, we have compared the effects of the acidified milk protein drink on muscle mass only with a carbohydrate drink, so further studies that compare acidified milk with other protein sources are necessary to confirm this finding.

Unlike the results on body composition, physical performance improved equally in both groups. In the MILK group, knee extension and flexion strength improved 10.3% and 11.1%, respectively, while lean body mass increased 1.5% during the intervention period. Not only muscle mass but also neuromuscular activity influences muscle strength [51]. Exercise is known to have beneficial effects on the neuromuscular junction through neuromuscular junction preservation, hypertrophy and sprouting [52, 53]. We propose that the effects of low-to-moderate intensity training on neuromuscular activity cause this physical performance improvement in both groups. We found that low-to-moderate intensity exercise training in the present study is a valuable method to improve physical performance in older adults, but low-dose milk protein supplementation did not result in significant benefits on physical performance.

Our intervention in the present study did not have a large effect on blood parameters, except for plasma uric acid levels. The acidified milk protein drink decreased plasma uric acid levels, and that may be a specific effect of milk protein. Several studies showed that skim milk or milk protein ingestion acutely decreased serum uric acid levels, but this did not apply to soy protein [54, 55]. A cross-sectional study also shows dairy consumption is associated with lower serum uric acid levels, but meat and seafood consumption are associated with higher serum uric acid levels [56]. Furthermore, there was a chronic urate-lowering effect of long-term milk protein ingestion in the present study. High blood uric acid levels, known as hyperuricemia, can lead to a disease called gout that causes painful joints that accumulate urate crystals. Long-term ingestion of milk protein would prevent hyperuricemia.

There are several limitations to the present study. First, the study design did not include a low-dose protein supplementation alone group, and that may limit the interpretation of the study results. It is not clear if the increase in muscle mass was due to low-dose protein supplementation alone or an interactive effect of protein supplementation and exercise although the 6-mo exercise alone did not result in an increase in muscle mass. Second, it is possible that amount of exercise performed over the intervention period in the present study might not be sufficient as opposed to what the participants reported. Krause et al. [33] showed an improvement in muscle mass over 12 weeks of a low-to-moderate intensity exercise intervention. The participants in Krause’s study performed a supervised exercise program whereas those in the present study were “instructed” to perform their exercises on their own. Insufficient amount of exercise compared to our instruction could be related to the fact that the low-to-moderate intensity exercise program used in the present study did not induce an increase in muscle mass. Finally, the participants in this study were healthy older adults whereas older adults often have co-morbidities, so the findings in this study should be interpreted carefully and applied only to healthy older adults. This healthy population may explain in part why we did not observe many changes in blood parameters.

In conclusion, the present study has demonstrated that long-term low-dose milk protein supplementation (10 g of protein/d) combined with low-to-moderate intensity exercise training is effective to increase muscle mass in healthy older adults. Furthermore, the intervention is associated with decreasing fat mass and plasma uric acid levels. However, during low-to-moderate intensity exercise training intervention, the low-dose milk protein supplementation did not have significant effects on muscle strength and function compared to isocaloric carbohydrate supplementation, so the physiological benefits of low-dose milk protein supplementation are limited. We also found that low-to-moderate intensity exercise training is a valuable method to improve physical performance in healthy older adults. Low-dose protein intake and low-to-moderate intensity exercise training are the key interventions in this study, and contribute to the low dropout rate and high compliance. We are encouraged that the nutrition and exercise strategies proposed in this study will improve the health of many older adults.

## Electronic supplementary material

Below is the link to the electronic supplementary material.

Supplementary file 1 (XLSX 98 kb)

## Data Availability

This manuscript has datasets included as electronic supplementary material.
